# Effects of the surface charge of polyamidoamine dendrimers on cellular exocytosis and the exocytosis mechanism in multidrug-resistant breast cancer cells

**DOI:** 10.1186/s12951-021-00881-w

**Published:** 2021-05-12

**Authors:** Jie Zhang, Mingjuan Li, Mingyue Wang, Hang Xu, Zhuoxiang Wang, Yue Li, Baoyue Ding, Jianqing Gao

**Affiliations:** 1 Department of Pharmaceutics, College of Medicine, Jiaxing University, Jiahang Road 118, Jiaxing, 314001 People’s Republic of China; 2 Department of Pharmacy, Shenyang Medical College, Wenhua Road 103, Shenyang, 110016 People’s Republic of China; 3 Institute of Pharmaceutics, College of Pharmaceutical Sciences, Zhejiang University, Room 409, Yuhangtang Road 866, 310058 Hangzhou, People’s Republic of China

**Keywords:** Surface charge, Exocytosis dynamics, Exocytosis pathways, ATP binding cassette proteins, Organelles, Major vault protein

## Abstract

**Background:**

Polyamidoamine (PAMAM) dendrimer applications have extended from tumor cells to multidrug-resistant tumor cells. However, their transportation in multidrug-resistant tumor cells remains unclear. Herein, we investigated the exocytosis rule and mechanism of PAMAM dendrimers in multidrug-resistant tumor cells.

**Results:**

Using a multidrug-resistant human breast cancer cell model (MCF-7/ADR), we performed systematic analyses of the cellular exocytosis dynamics, pathways and mechanisms of three PAMAM dendrimers with different surface charges: positively charged PAMAM-NH_2_, neutral PAMAM-OH and negatively charged PAMAM-COOH. The experimental data indicated that in MCF-7/ADR cells, the exocytosis rate was the highest for PAMAM-NH_2_ and the lowest for PAMAM-OH. Three intracellular transportation processes and P-glycoprotein (P-gp) participated in PAMAM-NH_2_ exocytosis in MCF-7/ADR cells. Two intracellular transportation processes, P-gp and multidrug resistance (MDR)-associated protein participated in PAMAM-COOH exocytosis. P-gp and MDR-associated protein participated in PAMAM-OH exocytosis. Intracellular transportation processes, rather than P-gp and MDR-associated protein, played major roles in PAMAM dendrimer exocytosis. PAMAM-NH_2_ could enter MCF-7/ADR cells by forming nanoscale membrane holes, but this portion of PAMAM-NH_2_ was eliminated by P-gp. Compared with PAMAM-OH and PAMAM-COOH, positively charged PAMAM-NH_2_ was preferentially attracted to the mitochondria and cell nuclei. Major vault protein (MVP) promoted exocytosis of PAMAM-NH_2_ from the nucleus but had no effect on the exocytosis of PAMAM-OH or PAMAM-COOH.

**Conclusions:**

Positive charges on the surface of PAMAM dendrimer promote its exocytosis in MCF-7/ADR cells. Three intracellular transportation processes, attraction to the mitochondria and cell nucleus, as well as nuclear efflux generated by MVP led to the highest exocytosis observed for PAMAM-NH_2_. Our findings provide theoretical guidance to design a surface-charged tumor-targeting drug delivery system with highly efficient transfection in multidrug-resistant tumor cells. Especially, to provide more DNA to the nucleus and enhance DNA transfection efficiency in multidrug-resistant tumor cells using PAMAM-NH_2_, siRNA-MVP or an inhibitor should be codelivered to decrease MVP-mediated nuclear efflux.

**Graphical abstract:**

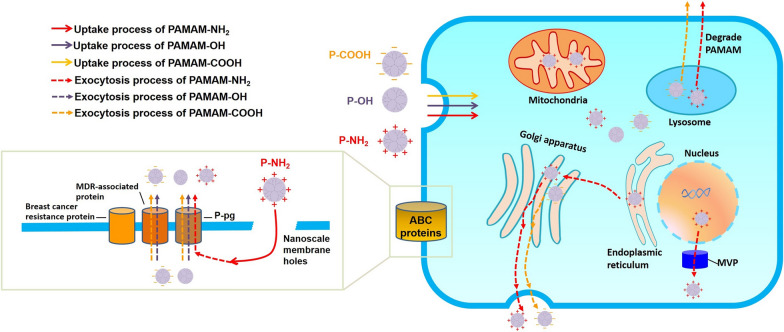

**Supplementary Information:**

The online version contains supplementary material available at 10.1186/s12951-021-00881-w.

## Background

Polyamidoamine (PAMAM) dendrimers are monodisperse, highly branched polymeric macromolecules with a uniform size and shape [1, 2]. Their basic structure includes three main components: a central core, repeated branching units, and terminal groups [1]. Because of their distinct structure, PAMAM dendrimers have steadily grown in popularity in the fields of drug delivery and gene therapy for antitumor treatment and the reversal of multidrug resistance (MDR) [3]. Antitumor drugs and targeting agents can be either encapsulated into internal PAMAM cavities or bound to their surfaces through hydrophobic or covalent bonds, which can be applied for targeted antitumor treatment [1, 4, 5]. Genes that downregulate the expression of MDR genes to restore drug sensitivity can be bound to PAMAM surfaces through electrostatic interactions, leading to the reversal of MDR [3, 6, 7].

The performance of PAMAM dendrimers is dependent upon their internal cavities and terminal groups. In particular, the numerous terminal functional groups of these dendrimers can not only deliver drugs and genes but also produce a highly localized charge density, namely, a positive charge, a negative charge or no charge, which can have a significant influence on the interactions of the dendrimer with the cell membrane and subsequent transportation in cells [2, 8]. Perumal et al. reported that the terminal groups influenced the cellular uptake pathway and distribution of PAMAM dendrimers in A549 cells [2]. Sadekar et al. reported that PAMAM dendrimers were transported by a combination of paracellular and transcellular routes in Caco-2 cells [9]. Other studies have reported that terminal amino groups on polyamidoamine (PAMAM-NH_2_) dendrimers can interact with the cell membrane and enter KB and Rat2 cells by forming nanoscale membrane holes but that other PAMAM dendrimers cannot [10, 11]. The influence of surface charge on the cellular uptake mechanism and subsequent intracellular transport may be dependent on the type of cell targeted. However, little information is available on the cellular trafficking of PAMAM dendrimers in resistant tumor cells. Our laboratory conducted studies on the transportation of the PAMAM-NH_2_ dendrimer in MCF-7/ADR cells [12]. We found that the uptake, intracellular distribution and exocytosis of the PAMAM-NH_2_ dendrimer in MCF-7 cells were different from those in MCF-7/ADR cells. We speculated that the charges on the surface of the PAMAM-NH_2_ dendrimer contributed to these differences. Dendrimer surface charges could lead to toxicity [1, 5, 13], and cancer cells could become resistant to the cytotoxic effects of various structurally and mechanistically unrelated chemotherapeutic agents owing to MDR, so resistant tumor cells may show MDR against PAMAM dendrimers with different surface charges. One of the MDR mechanisms is increasing drug influx [14–16], so the exocytosis of PAMAM dendrimers with different surface charges may be different in resistant tumor cells.

To investigate the effects of the PAMAM dendrimer surface charge on the exocytosis and exocytosis mechanism in multidrug-resistant tumor cells, we used multidrug-resistant human breast cancer cells (MCF-7/ADR cells) as a cell model. We performed systematic analyses of the cellular exocytosis dynamics, pathways and mechanisms, including the effects of ATP binding cassette (ABC) proteins, organelles and major vault protein (MVP) on the exocytosis of three PAMAM dendrimers with different surface charges, namely, the positively charged PAMAM-NH_2_ dendrimer, the neutral PAMAM-OH dendrimer, and the negatively charged PAMAM-COOH dendrimer. Our findings provide theoretical guidance for designing nanocarriers with surface charge-mediated tumor-targeted drug delivery systems in multidrug-resistant tumor cells.

## Results and discussion

### Stabilities of the PAMAM-FITC dendrimers at different pH values

Tumors constantly remain an acidic pH (often in the range of 6.5–6.8), while the pH in endosomes/lysosomes is 5.0 [17, 18]. Our previous research indicated that a portion of PAMAM dendrimers could be internalized into MCF-7/ADR cells through endocytosis and then trapped in endosomes/lysosomes [12] and that FITC may dissociate from the surface of the PAMAM dendrimers during this process. Therefore, it was necessary to investigate the stabilities of the PAMAM-FITC dendrimers at both pH 6.8 and pH 5.0. Table 1 shows that the fluorescence intensities of the PAMAM-NH_2_, PAMAM-OH and PAMAM-COOH dendrimers decreased after 12 h of dialysis in pH 6.8 and pH 5.0 phosphate-buffered saline (PBS), but the change rates remained below 20%. These data indicate that the PAMAM-FITC dendrimers were stable at pH 6.8 and pH 5.0; the green fluorescence observed in this study represents the PAMAM dendrimers rather than free FITC.

**Table 1:** The fluorescence intensity change rates of the PAMAM dendrimers at different pH values

	pH 6.8	pH 5.0
PAMAM-NH_2_-FITC	11.74%	12.63%
PAMAM-OH-FITC	10.34%	18.56%
PAMAM-COOH-FITC	10.78%	15.25%

### Cytotoxicity of the PAMAM dendrimers

**Fig. 1:**
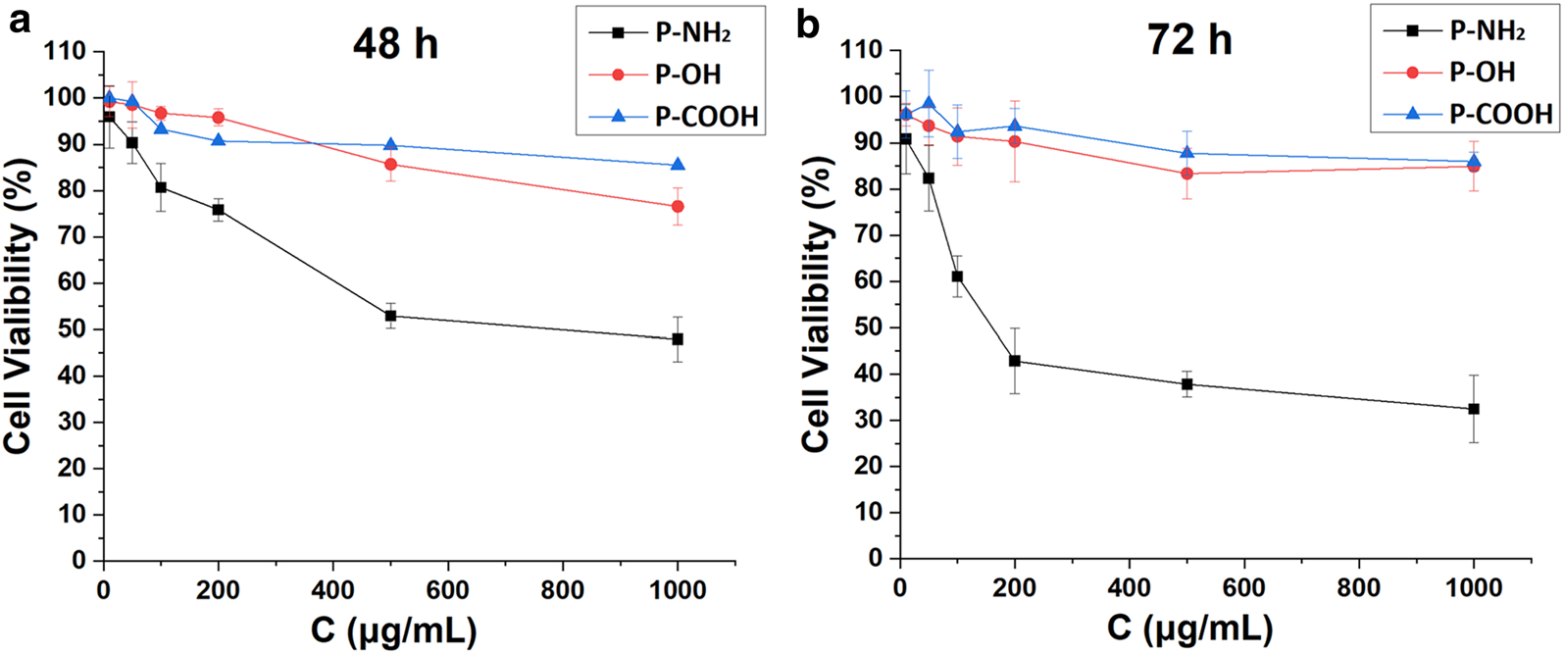
Evaluation of the cytotoxicity of the PAMAM dendrimers after 48 h (**a**) and 72 h (**b**). Mean ± SD, n = 6

Cell viabilities at different concentrations of PAMAM-NH_2_, PAMAM-OH and PAMAM-COOH were used to investigate the cytotoxicity of these dendrimers in vitro. As shown in Fig. 1, at low concentrations (1–50 µg/mL), the viabilities of MCF-7/ADR cells treated with all three PAMAM dendrimers after 48 and 72 h were more than 80%, indicating low cytotoxicity against MCF-7/ADR cells. As the concentration increased, the viability of the MCF-7/ADR cells decreased sharply after treatment with the PAMAM-NH_2_ dendrimer, while MCF-7/ADR cell viability remained above 80% after treatment with the PAMAM-OH and PAMAM-COOH dendrimers. After treatment with 1000 µg/mL PAMAM-NH_2_ dendrimer for 72 h, the viability of the MCF-7/ADR cells decreased to 32.48 ± 7.3%. These results revealed that the PAMAM-OH and PAMAM-COOH dendrimers were not cytotoxic to MCF-7/ADR cells, while the PAMAM-NH_2_ dendrimer showed concentration-dependent toxicity. As reported previously, the positively charged amine groups on the surface of PAMAM-NH_2_ can interact with negatively charged cell membranes, damaging these cell membranes and causing cell lysis [8]. The results obtained in this study were consistent with those of previous reports [12, 19]. In this study, the only difference between the PAMAM-NH_2_, PAMAM-OH and PAMAM-COOH dendrimers is their surface charge, so the concentration-dependent toxicity of the PAMAM-NH_2_ dendrimer is due to its cationic surface groups. Notably, the PAMAM dendrimers at a concentration of 20 µg/mL were not toxic to MCF-7/ADR cells and could therefore be used in other studies in this report.

### Time dependence of cellular exocytosis

**Fig. 2:**
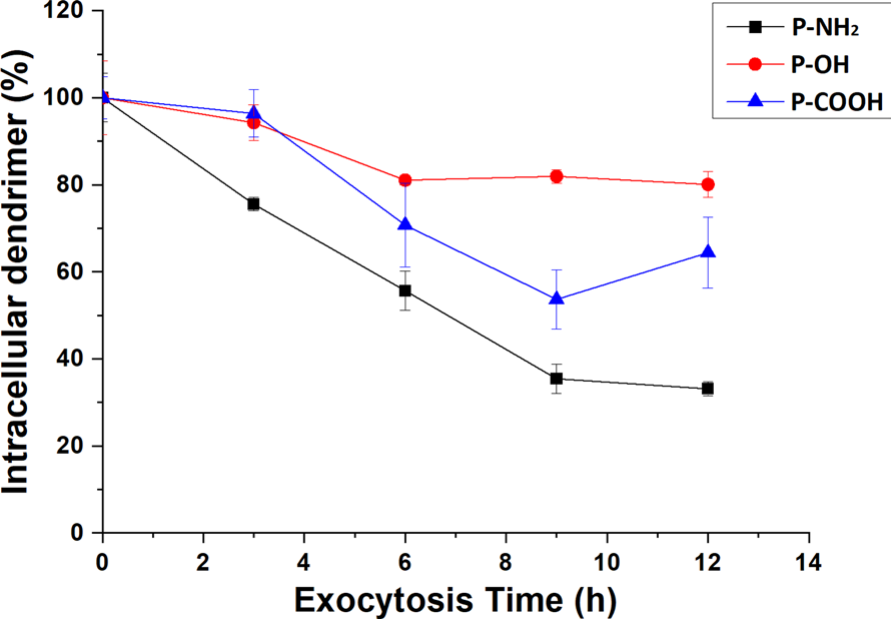
Intracellular PAMAM dendrimer content at different exocytosis times in MCF-7/ADR cells. All values are relative to the amount at 0 h. Mean ± SD, n = 3

To investigate the exocytosis dynamics, we detected the intracellular fluorescence intensity of the PAMAM-NH_2_, PAMAM-OH and PAMAM-COOH dendrimers at different time points (3, 6, 9 and 12 h) after removal of the PAMAM-NH_2,_ PAMAM-OH and PAMAM-COOH dendrimers. To investigate the cellular exocytosis of these dendrimers by MCF-7/ADR cells, we ensured that approximately 30% of the amine, hydroxyl radical and carboxyl groups on the surfaces of the PAMAM dendrimers were modified with FITC. As shown in Fig. 2; Table 2, from 0 to 9 h, the intracellular PAMAM-NH_2_ dendrimer content sharply decreased to 35.45 ± 3.39%. In contrast, from 9 to 12 h, the amount of intracellular PAMAM-NH_2_ dendrimer in MCF-7/ADR cells declined steadily, from 35.45 ± 3.39% to 33.11 ± 1.6%, which suggested that the exocytosis of the PAMAM-NH_2_ dendrimer reached equilibrium after 9 h. The intracellular PAMAM-OH dendrimer content declined steadily and was maintained at approximately 80% after 6 h, which suggested that the exocytosis of the PAMAM-OH dendrimer reached equilibrium after 6 h. Exocytosis of the PAMAM-COOH dendrimer took place after 3 h. From 3 h to 9 h, the intracellular content of the PAMAM-COOH dendrimer sharply decreased, similar to that observed for the PAMAM-NH_2_ dendrimer. Although the intracellular content of the PAMAM-COOH dendrimer increased at 12 h, the amount of intracellular PAMAM-COOH dendrimer between 9 and 12 h was not significantly different (*P* > 0.05). Thus, exocytosis of the PAMAM-COOH dendrimer reached equilibrium after 9 h, and the intracellular content of this dendrimer was maintained at approximately 60%. It has been reported that nonspecific binding would be expected due to ionic interactions. The cations on the surface of the PAMAM-NH_2_ dendrimer can bind with proteoglycans on the cell membrane. Seib et al. reported that low temperatures can block internalization and do not influence external binding at the surface. Thus, the cellular uptake at 4 °C can represent the amount of external binding [20]. We detected the cellular uptake rates of the PAMAM-NH_2_, PAMAM-OH and PAMAM-COOH dendrimers into MCF-7/ADR cells at 4 °C (Additional file 1: Fig. S1). Additional file 1: Fig. S1 shows that the uptake rates of the PAMAM-NH_2_, PAMAM-OH and PAMAM-COOH dendrimers remained below 5%, revealing that the extracellular binding of PAMAM dendrimers to the MCF-7/ADR cell surface could be removed through the cell sample treatment method in this study and that the intracellular fluorescence intensity tested represents the intracellular content of the PAMAM dendrimers. Overall, the exocytosis rates from MCF-7/ADR cells in decreasing order were PAMAM-NH_2_ > PAMAM-COOH > PAMAM-OH, which suggested that a charge on the surface of the carrier could increase exocytosis in MCF-7/ADR cells. The highest degree of exocytosis from MCF-7/ADR cells observed for the PAMAM-NH_2_ dendrimer should contribute to the following points. First, the MCF-7/ADR cells used in this study show significant MDR (as displayed in Additional file 1: Table S1 and Fig. S2). Cancer cells become resistant to the cytotoxic effects of various structurally and mechanistically unrelated chemotherapeutic agents owing to MDR [16, 21]. Second, we indicated that the PAMAM-NH_2_ dendrimer showed concentration-dependent toxicity against MCF-7/ADR cells. Therefore, MCF-7/ADR cells may develop resistance to the PAMAM-NH_2_ dendrimer. Another major mechanism of MDR is increased drug efflux, which leads to the highest degree of PAMAM-NH_2_ exocytosis from MCF-7/ADR cells.

**Table 2:** Intracellular PAMAM dendrimer content at different exocytosis times in MCF-7/ADR cells

	0 h	3 h	6 h	9 h	12 h
PAMAM-NH_2_	100 ± 5.5%	75.54 ± 1.5%	55.67 ± 4.5%	35.45 ± 3.4%	33.11 ± 1.6%
PAMAM-OH	100 ± 8.5%	94.25 ± 4.1%	81.04 ± 1.1%	81.93 ± 1.6%	80.08 ± 2.9%
PAMAM-COOH	100 ± 4.9%	96.37 ± 5.4%	70.73 ± 9.5%	53.66 ± 6.8%	64.39 ± 8.1%

All values are relative to the amount at 0 h. Mean ± SD, n = 3

### Influence of intracellular transport inhibitors on PAMAM dendrimer exocytosis

**Fig. 3:**
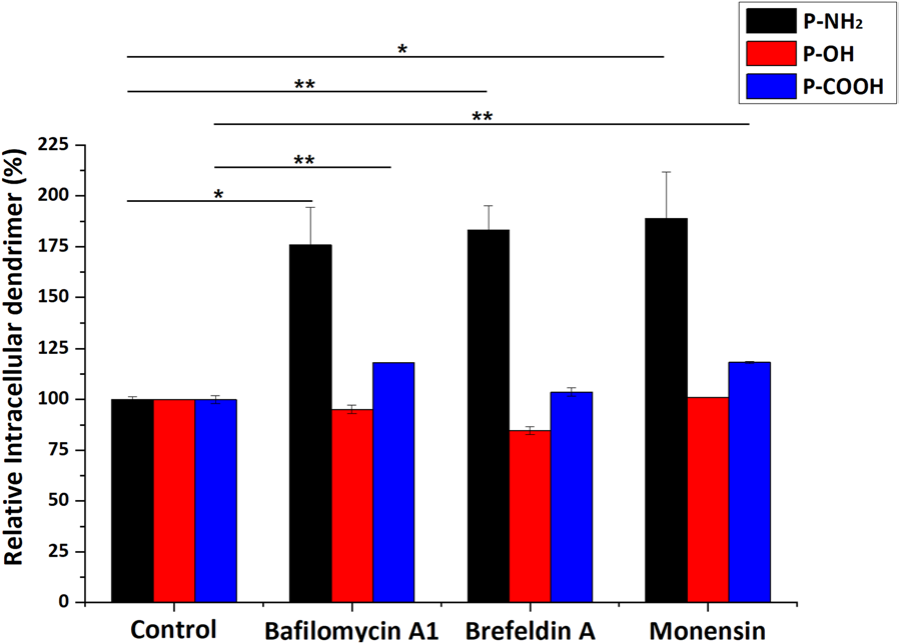
The effects of different intracellular transport inhibitors on the exocytosis of PAMAM dendrimers. The control samples were cells without inhibitor pretreatment. All values are relative to the control. * indicates *P* < 0.05 vs. the control group, ** indicates *P* < 0.01 vs. the control group; mean ± SD, n = 3

Exocytosis is a common process by which materials are exported from a biological cell. In our study, we estimated the remaining fluorescence signals in the cell, which included degradation in addition to exocytosis [22]. Bafilomycin A1, an inhibitor of acidification, may prevent macromolecules from being transported to lysosomes, resulting in the avoidance of degradation. Brefeldin A blocks transport from the endoplasmic reticulum (ER) to the Golgi complex. Monensin blocks transport from the Golgi complex to the plasma membrane [22–24]. As shown in Fig. 3, the amount of remaining PAMAM-NH_2_ dendrimer in MCF-7/ADR cells significantly increased with the addition of bafilomycin A1, brefeldin A and monensin, indicating that endosomal acidification increased the degradation of PAMAM-NH_2_ and that two processes, namely, transportation from the ER to the Golgi complex and transportation from the Golgi complex to the plasma membrane, participated in PAMAM-NH_2_ dendrimer exocytosis from MCF-7/ADR cells. The remaining PAMAM-OH dendrimer content did not increase with the addition of bafilomycin A1, brefeldin A or monensin, indicating that endosomal acidification, the Golgi complex or the ER did not participate in PAMAM-OH dendrimer exocytosis. The remaining PAMAM-COOH dendrimer content significantly increased with the addition of bafilomycin A1 and monensin, suggesting that endosomal acidification led to an increase in the degradation of the PAMAM-COOH dendrimer and that transportation from the Golgi complex to the plasma membrane participated in PAMAM-COOH dendrimer exocytosis from MCF-7/ADR cells. Therefore, we concluded that the three processes, namely, endosomal acidification, transportation from the ER to the Golgi complex and transportation from the Golgi complex to the plasma membrane, participated in the exocytosis of the PAMAM-NH_2_ dendrimer from MCF-7/ADR cells. Two processes, namely, endosomal acidification and transportation from the Golgi complex to the plasma membrane, participated in the exocytosis of the PAMAM-COOH dendrimer in MCF-7/ADR cells. The PAMAM-OH dendrimer was hardly eliminated from MCF-7/ADR cells through the above three processes. These results led to different amounts of the PAMAM-NH_2_, PAMAM-OH and PAMAM-COOH dendrimers being excreted by MCF-7/ADR cells (Fig. 2).

### Influence of ABC protein inhibitors on PAMAM dendrimer exocytosis

**Fig. 4:**
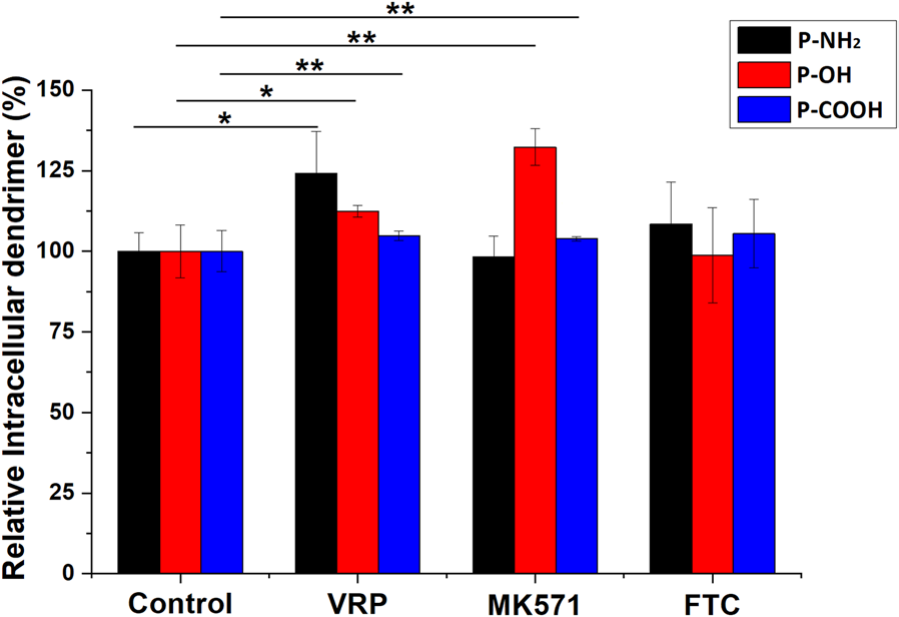
The effects of different ABC transporter inhibitors on the exocytosis of PAMAM dendrimers. The control samples were cells without inhibitor pretreatment. All values are relative to the control. * indicates *P* < 0.05 vs. the control group, ** indicates *P* < 0.01 vs. the control group; mean ± SD, n = 3

The overexpression of ABC proteins, such as P-glycoprotein (P-gp), MDR-associated protein and breast cancer resistance protein, which can increase drug efflux, is one of the MDR mechanisms in multidrug-resistant cells [3, 25]. To investigate the influence of these ABC proteins on the exocytosis of PAMAM dendrimers, we used VRP, MK571 and FTC as inhibitory agents (see Table 3), which can inhibit the expression of P-gp, MDR-associated protein and breast cancer resistance protein, respectively [25–27]. As shown in Fig. 4, compared with the controls, the remaining PAMAM-NH_2_ dendrimer in MCF-7/ADR cells significantly increased with the addition of VRP, indicating that the PAMAM-NH_2_ dendrimer could be repulsed by P-gp. The remaining PAMAM-OH and PAMAM-COOH dendrimers in MCF-7/ADR cells significantly increased with the addition of VRP and MK571, indicating that the PAMAM-OH and PAMAM-COOH dendrimers could be repulsed by both P-gp and MDR-associated protein.

The results in Fig. 3 indicate that intracellular transport did not participate in exocytosis of the PAMAM-OH dendrimer. Therefore, the PAMAM-OH dendrimer could be eliminated by only P-gp and MDR-associated protein. The results in Fig. 2 show that the amount of exocytosis of the PAMAM-OH dendrimer was the lowest. These results suggest that the degree of exocytosis of the PAMAM-OH dendrimer repelled by P-gp and MDR-associated protein was low. Moreover, compared with the PAMAM-NH_2_ and PAMAM-COOH dendrimer groups in Figs. 3 and 4, the amounts of remaining PAMAM-NH_2_ and PAMAM-COOH dendrimers after the addition of the intracellular transportation inhibitor were higher than those after the addition of P-gp and MDR-associated protein. These results suggested that intracellular transportation rather than P-gp and MDR-associated protein was the major PAMAM dendrimer exocytosis pathway.

Earlier reports have suggested that nanocarriers can bypass drug efflux by ABC proteins, as nanocarriers are internalized via either nonspecific or specific endocytosis to overcome MDR [3, 14, 15]. However, the results of the current study show that P-gp and MDR-associated protein could eliminate PAMAM dendrimers, which is contrary to previous reports. Additionally, some reports have indicated that in addition to endocytosis, dendrimers can enter cells through the energy-dependent formation of nanoscale membrane holes [10, 11]. It was therefore hypothesized that this portion of the PAMAM dendrimers may not bypass ABC proteins.

### Lactate dehydrogenase (LDH), propidium iodide (PI) and fluorescein diacetate (FDA) assay

**Fig. 5:**
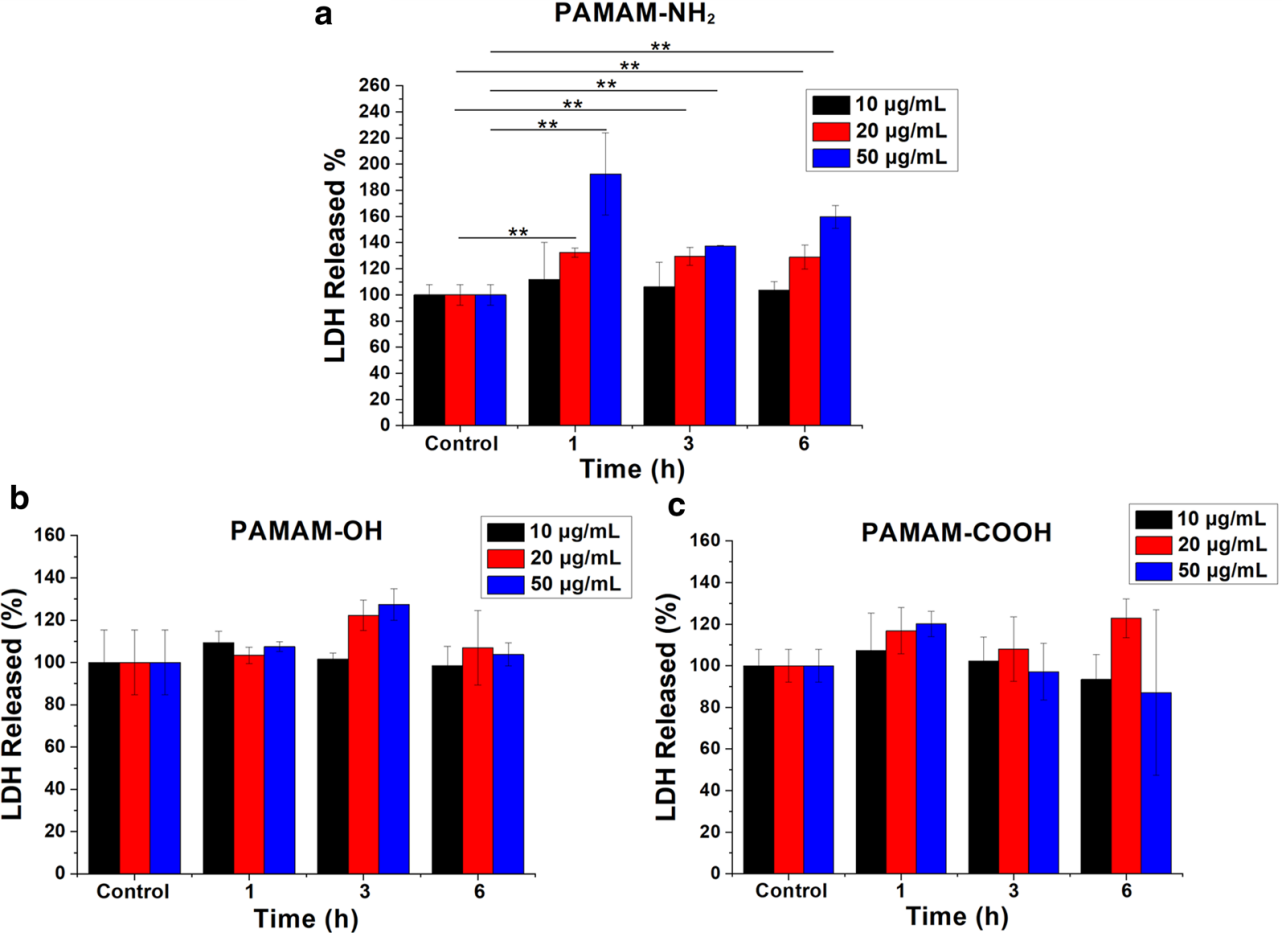
The released amount of LDH after PAMAM-NH_2_ (**a**), PAMAM-OH (**b**) and PAMAM-COOH (**c**) dendrimer treatment. The control samples were cells without PAMAM dendrimer treatment. All values are relative to the control. ** indicates *P* < 0.01 vs. the control group; mean ± SD, n = 6

**Fig. 6:**
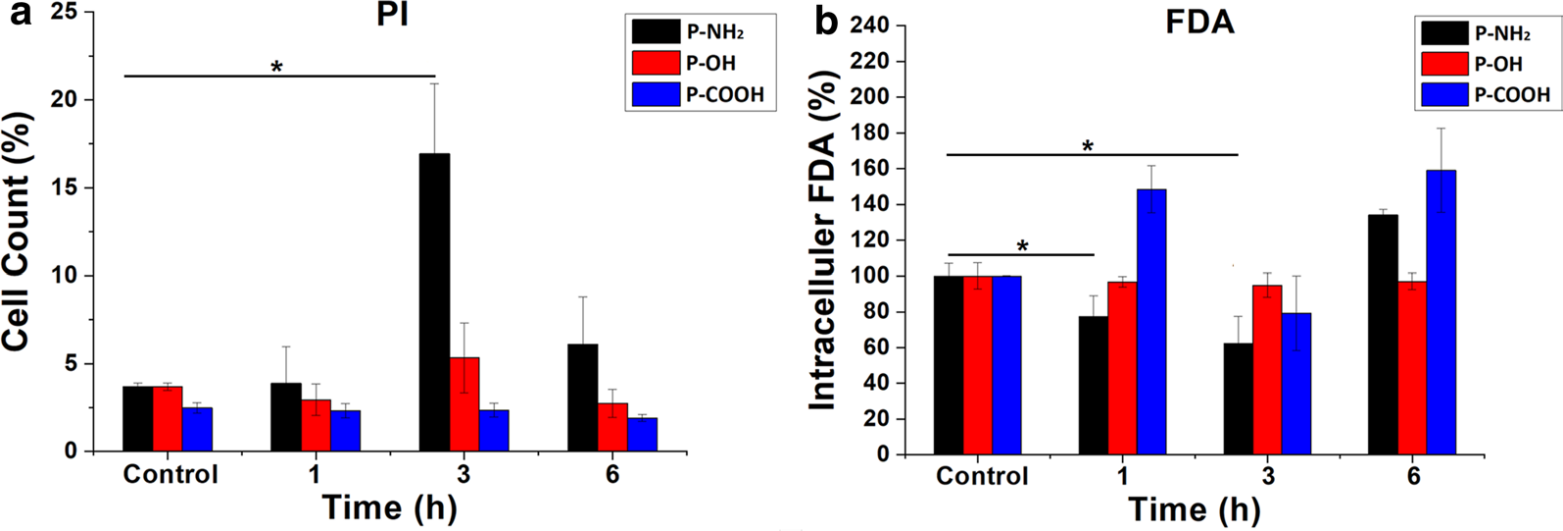
The amount of PI (**a**) and FDA (**b**) in MCF-7/ADR cells after PAMAM dendrimer treatment. The control samples were cells without PAMAM dendrimer treatment. All values in **b** are relative to the control. * indicates P < 0.05 vs. the control group; mean ± SD, n = 3

To investigate whether the PAMAM dendrimers could enter MCF-7/ADR cells through the energy-dependent formation of nanoscale membrane holes, we detected the cellular leakage of LDH and intracellular fluorescence intensity with the addition of PI and FDA. As reported, intracellular LDH is released when cell membrane permeability increases [10, 28]. PI is readily internalized into cells with disrupted membranes but is excluded from cells with intact membranes [28, 29]. FDA is a nonfluorescent compound that readily enters intact cells and then undergoes hydrolysis by endogenous esterases, resulting in the release of fluorescein into the cytosol. Cytosolic fluorescein is not able to transfer through a normal cell membrane without holes [28, 29]. Figure 5 shows that compared with the control, the extracellular LDH content significantly increased following treatment with the PAMAM-NH_2_ dendrimer but extracellular LDH did not display a significant change following treatment with the PAMAM-OH and PAMAM-COOH dendrimers. Figure 6 shows that compared with the controls, the content of intracellular PI significantly increased and the content of intracellular FDA significantly decreased after 3 h of treatment with the PAMAM-NH_2_ dendrimer, while the contents of intracellular PI and FDA did not change after incubation with the PAMAM-OH and PAMAM-COOH dendrimers from 1 to 6 h. The results in Figs. 5 and 6 suggest that only the PAMAM-NH_2_ dendrimer could enhance cell membrane permeability. As previously reported, cell death leads to an increase in membrane permeability [30–32]. The concentration of the PAMAM-NH_2_ dendrimer used in this study was 20 µg/mL, and we showed that low concentrations (1–50 µg/mL) of this dendrimer showed no cytotoxicity to MCF-7/ADR cells. Thus, the observed change in cell membrane permeability resulted from nanoscale membrane holes formed by the uptake of the PAMAM-NH_2_ dendrimer rather than apoptosis. Only the PAMAM-NH_2_ dendrimer, not the PAMAM-OH or PAMAM-COOH dendrimer, could induce nanoscale membrane holes on the cytomembrane and enter MCF-7/ADR cells through these holes. The surfaces of the PAMAM-NH_2_, PAMAM-OH and PAMAM-COOH dendrimers possess positive charges, negative charges and no charge, respectively. Leroueil et al. reported that only polycationic nanoparticles could induce the production of nanoscale membrane holes on the membrane and enter cells [11]. Thus, only the PAMAM-NH_2_ dendrimer could enter MCF-7/ADR cells by forming nanoscale membrane holes on the cytomembrane, and this portion of the PAMAM-NH_2_ dendrimer could be eliminated by P-gp. Figure 4 shows that the PAMAM-OH and PAMAM-COOH dendrimers could also be eliminated by P-gp and MDR-associated protein and cannot enter MCF-7/ADR cells by forming nanoscale membrane holes on the cytomembrane; thus, PAMAM-OH and PAMAM-COOH dendrimers may have other interactions with P-gp and MDR-associated protein, which we are investigating.

### Colocalization with organelles in MCF-7/ADR cells

**Fig. 7:**
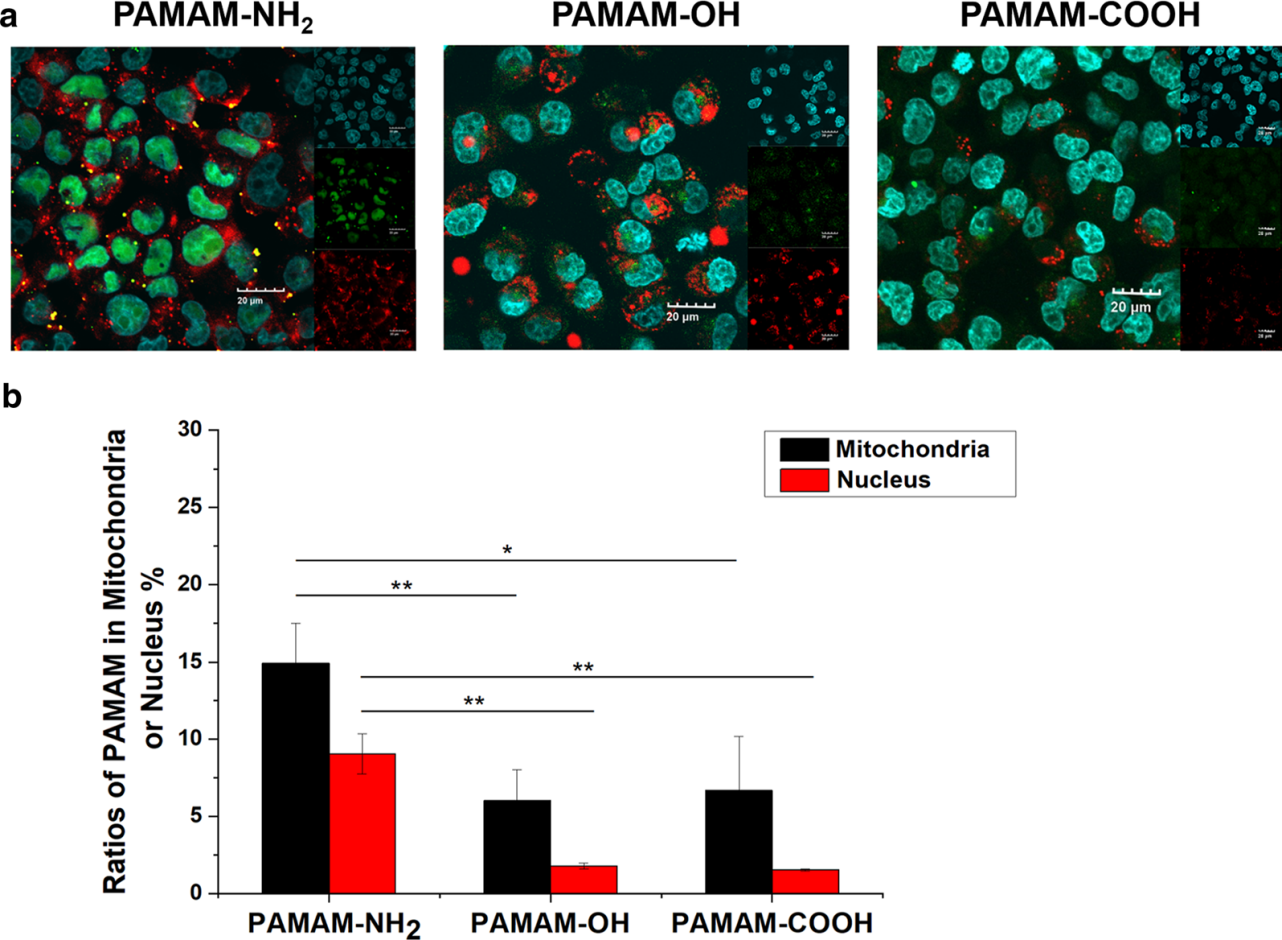
Cellular uptake confocal images of the distribution of PAMAM dendrimers in MCF-7/ADR cells (**a**). The ratios of PAMAM dendrimers in the mitochondria and nuclei of MCF-7/ADR cells (**b**). PAMAM dendrimers are green, the nuclei are stained blue using Hoechst 33258, and the mitochondria are stained red with MitoTracker Red. The scale bar is 20 μm. * indicates *P* < 0.05 vs. the PAMAM-NH_2_ group, ** indicates *P* < 0.01 vs. the PAMAM-NH_2_ group; mean ± SD, n = 3

The distribution of nanoparticles into organelles is a key factor in exocytosis [33]. Additionally, the mitochondria and cell nuclei are major targets of antitumor drugs. Therefore, we detected the distributions of the PAMAM-NH_2_, PAMAM-OH and PAMAM-COOH dendrimers in the mitochondria and cell nuclei by laser scanning confocal microscopy (LSCM) and fluorescence spectrophotometry. As shown in Fig. 7a, the cells were labeled with a dye selective for mitochondria (MitoTracker Red, red) and a nucleus-selective dye (Hoechst 33,258, blue). Bright green fluorescence due to aggregation of the PAMAM-NH_2_ dendrimer was found in the MCF-7/ADR cell cytoplasm, which overlapped with the red fluorescence in MCF-7/ADR cells, indicating that some of the PAMAM-NH_2_ dendrimers aggregated in the mitochondria of MCF-7/ADR cells. In contrast, the green fluorescence from the PAMAM-OH and PAMAM-COOH dendrimers was evenly distributed in the MCF-7/ADR cell cytoplasm. These results indicated that the PAMAM-NH_2_ dendrimer, but not the PAMAM-OH or PAMAM-COOH dendrimer, could enter the mitochondria of MCF-7/ADR cells. Figure 7a also shows that some of the green fluorescence from the PAMAM-NH_2_ dendrimer overlapped with the blue fluorescence from the nuclei in the MCF-7/ADR cells, but this phenomenon did not appear in the PAMAM-OH and PAMAM-COOH groups, which indicated that the PAMAM-NH_2_ dendrimer could also enter the MCF-7/ADR cell nucleus.

We isolated the mitochondria and nuclei of MCF-7/ADR cells and detected the fluorescence intensities inside them. Figure 7b shows that the ratios of the PAMAM-NH_2_ dendrimer in the mitochondria and nuclei were significantly higher than the ratios of the PAMAM-OH and PAMAM-COOH dendrimers in the mitochondria and nuclei, which further suggested that compared with the PAMAM-OH and PAMAM-COOH dendrimers, the PAMAM-NH_2_ dendrimer showed greater distribution into the mitochondria and nuclei of MCF-7/ADR cells. These results are consistent with the results in Fig. 7a. Compared with other organelles, the electron transport chain and ATP synthesis via oxidative phosphorylation create a membrane electrochemical gradient that leads to a high membrane potential of 180–200 mV in the mitochondria, which results in the preferential attraction of cationic molecules [34, 35]. Therefore, compared with the PAMAM-OH and PAMAM-COOH dendrimers, the positively charged PAMAM-NH_2_ dendrimer was preferentially distributed in the mitochondria of MCF-7/ADR cells. Brunner et al. reported that the diameter of nuclear pores is 9–10 nm and that molecules less than 10 nm in diameter could be transported into the nucleus through these pores [36]. The PAMAM dendrimers used in this study are approximately 5 nm in diameter, and the positively charged PAMAM-NH_2_ dendrimer can combine with DNA in vitro and has been widely used as a nonviral gene vector. Thus, the positively charged PAMAM-NH_2_ dendrimer was preferentially distributed in the nucleus of MCF-7/ADR cells compared with the other PAMAM dendrimers. Sakhtianchi et al. reported that nanoparticles that are encapsulated by a membrane in the cytoplasm are more easily excreted by cells than unencapsulated nanoparticles [33]. Thus, the PAMAM-NH_2_ dendrimer, which was preferentially distributed in the mitochondria and nucleus, was more easily excreted. These results also suggested that compared with PAMAM-OH and PAMAM-COOH dendrimers, the PAMAM-NH_2_ dendrimer can be used as a highly effective mitochondria-targeting agent and gene drug carrier for multidrug-resistant tumor therapy.

### Influence of MVP on the exocytosis of PAMAM dendrimers

**Fig. 8:**
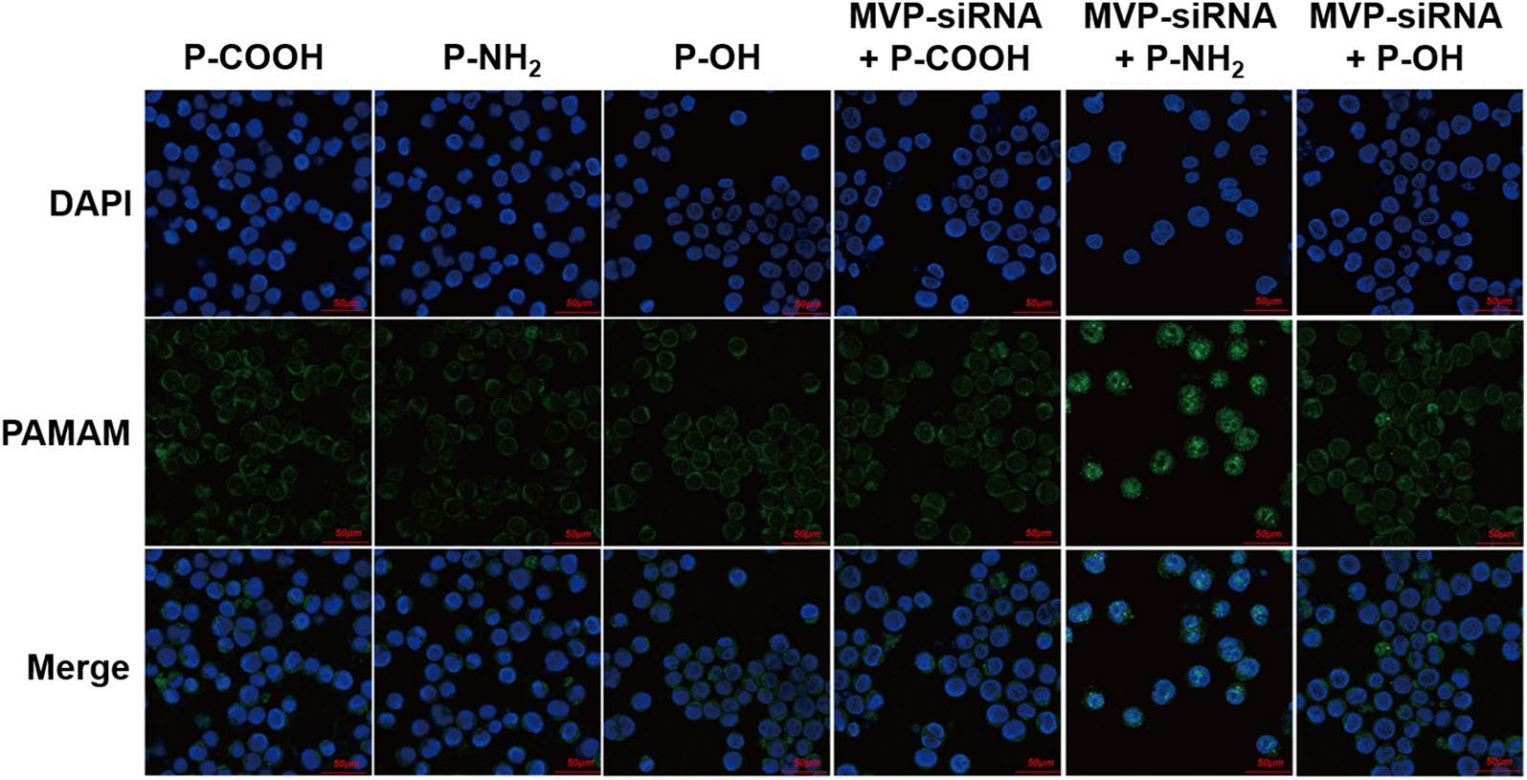
Cellular uptake confocal images of the distribution of PAMAM dendrimers in MCF-7/ADR cells. PAMAM dendrimers are green while the nuclei are stained blue using Hoechst 33258. The scale bar is 50 μm

**Fig. 9:**
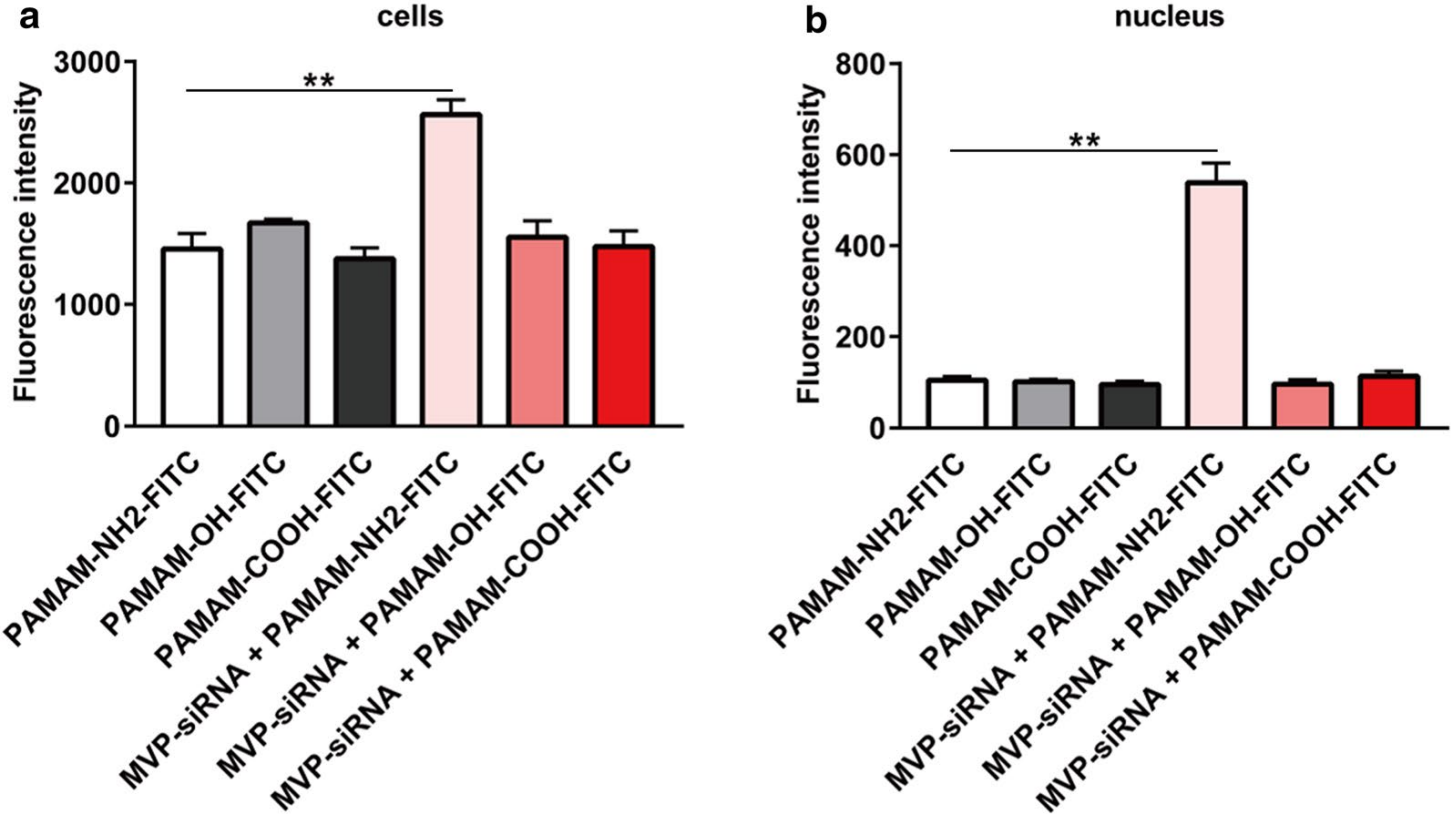
The fluorescence intensity of PAMAM dendrimers in MCF-7/ADR cells (**a**) and the nuclei in MCF-7/ADR cells (**b**). ** indicates *P* < 0.01 vs. the PAMAM-NH_2_-FITC group; mean ± SD, n = 3

MVP, with a molecular weight (MW) of 110 kDa, is abundantly present in the cytoplasm of eukaryotic cells and participates in intracellular transport [7, 37]. To determine whether MVP participated in the transport of PAMAM dendrimers in MCF-7/ADR cells, we used siRNA-MVP to downregulate the expression of MVP and detected the amount of remaining intracellular PAMAM dendrimers after 3 h of exocytosis. As shown in Fig. 8, in the absence of siRNA-MVP, the green fluorescence of the PAMAM-NH_2_ dendrimer was located in the cytoplasm and did not overlap with the nucleus, which indicated that the PAMAM-NH_2_ dendrimer was eliminated from the nucleus after 3 h of exocytosis. After the addition of siRNA-MVP, the green fluorescence of the PAMAM-NH_2_ dendrimer again overlapped with the blue fluorescence of the nucleus, and the green fluorescence intensity increased. However, the distribution and intensity of the green fluorescence from PAMAM-OH and PAMAM-COOH dendrimers did not change after the addition of siRNA-MVP. These results indicated that MVP participated in the nucleo-cytoplasmic transport of the PAMAM-NH_2_ dendrimer and promoted the elimination of the PAMAM-NH_2_ dendrimer from the nucleus of MCF-7/ADR cells. The quantitative detection results are shown in Fig. 9. Figure 9b shows that after the addition of siRNA-MVP, the fluorescence intensity of the PAMAM-NH_2_ dendrimer in the nuclei of MCF-7/ADR cells significantly increased, while the fluorescence intensities of the PAMAM-OH and PAMAM-COOH dendrimers in the nuclei of MCF-7/ADR cells did not change, further suggesting that MVP could facilitate elimination of the PAMAM-NH_2_ dendrimer, rather than the PAMAM-OH and PAMAM-COOH dendrimers, from the MCF-7/ADR cell nuclei. Figure 9a shows that the intracellular fluorescence intensity of the PAMAM-NH_2_ dendrimer significantly increased after the addition of siRNA-MVP, which indicated that elimination of the PAMAM-NH_2_ dendrimer by MVP could facilitate exocytosis of the PAMAM-NH_2_ dendrimer, but not the PAMAM-OH or PAMAM-COOH dendrimer, from MCF-7/ADR cells. Therefore, elimination of the PAMAM-NH_2_ dendrimer by MVP was another factor contributing to the highest extent of exocytosis from MCF-7/ADR cells observed for the PAMAM-NH_2_ dendrimer among the dendrimers tested. It was therefore hypothesized that this phenomenon may contribute to the resistance of MCF-7/ADR cells. MVP is overexpressed in MCF-7/ADR cells and participates in nucleo-cytoplasmic transport, especially transporting drugs away from their cellular targets, e.g., the nucleus, which has been proposed to be related to MDR [7]. We have indicated that the PAMAM-NH_2_ dendrimer showed cytotoxicity to MCF-7/ADR cells and that MCF-7/ADR cells may develop resistance to the PAMAM-NH_2_ dendrimer. Thus, MVP, which is related to MDR, influenced the exocytosis of the PAMAM-NH_2_ dendrimer but not the PAMAM-OH or PAMAM-COOH dendrimer. The above phenomenon suggests that when using the PAMAM-NH_2_ dendrimer to deliver DNA, we should co-deliver siRNA-MVP to provide more DNA to the nucleus and enhance the DNA transfection efficiency.

## Conclusions

**Fig. 10:**
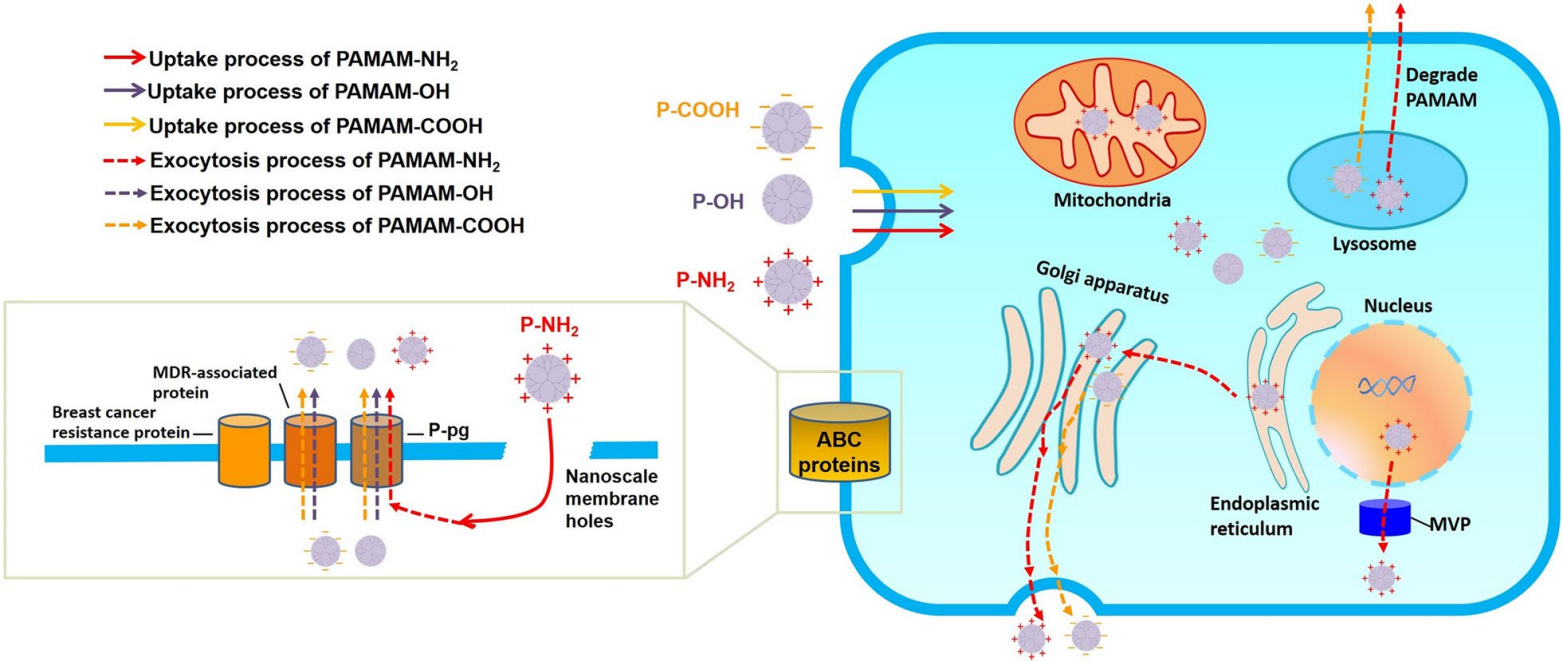
Schematic diagram showing the exocytosis processes (dotted arrow) of PAMAM-NH_2_ (red arrow), PAMAM-OH (purple arrow) and PAMAM-COOH (yellow arrow) dendrimers from MCF-7/ADR cells

In summary, a positive charge on the surface of PAMAM dendrimers promotes exocytosis in MCF-7/ADR cells, while neutral and negatively charged dendrimers have little effect on exocytosis. Positively charged PAMAM-NH_2_, which prefers to distribute into the mitochondria and nuclei, is an ideal mitochondria-targeting agent and gene drug carrier for multidrug-resistant tumor therapy. The three intracellular transportation processes examined increased dendrimer distribution in the mitochondria and the cell nucleus, as well as the nuclear efflux generated by MVP led to the highest exocytosis of PAMAM-NH_2_. Our findings regarding exocytosis rule and the mechanism of PAMAM dendrimers in MCF-7/ADR cells (Fig. 10) provide theoretical guidance for designing a surface-charged tumor-targeting drug delivery system with highly efficient transfection in multidrug-resistant tumor cells. In particular, to deliver greater amounts of DNA to the nucleus in multidrug-resistant tumor cells using PAMAM-NH_2_, we suggest the codelivery of siRNA-MVP or an inhibitor to decrease the nuclear efflux generated by MVP.

## Materials and methods

### Materials

PAMAM-G4-NH_2_, PAMAM-G4-OH, PAMAM-G4-COOH and doxorubicin (DOX) were purchased from Sigma-Aldrich (St. Louis, MO, USA). RPMI 1640 growth medium, fetal bovine serum (FBS) and penicillin-streptomycin were purchased from Gibco BRL (Gaithersburg, MD, USA). Methylthiazolyldiphenyl-tetrazolium bromide (MTT), a cell mitochondria isolation kit, Hoechst 33258, a 4′,6-diamidino-2-phenylindole (DAPI) LDH analysis kit and PI were purchased from Beyotime Biotechnology Co., Ltd. (Nantong, China). A cell nucleus isolation kit was purchased from KeyGEN Biotechnology Co., Ltd. (Nanjing, China). MitoTracker Red dye was purchased from Thermo Fisher Scientific (USA). siRNA-MVP was purchased from GenePharm (Shanghai, China). FDA was purchased from AAT Bioquest (USA). All other chemicals were commercially available reagents of at least analytical grade.

### Cell culture

MCF-7/ADR cells obtained from professor Jianqing Gao’s laboratory were maintained in 1640 growth medium supplemented with 10% FBS, penicillin (100 U/mL)-streptomycin (100 µg/mL) and DOX (1 µg/mL). The cells were incubated at 37 °C in a humidified atmosphere containing 5% CO_2_.

### Synthesis of FITC-labeled dendrimers

#### FITC-labeled PAMAM-G4-NH_2_ dendrimer

The PAMAM-G4-NH_2_ dendrimer was labeled with FITC as previously described [38]. A solution of FITC solution in acetone was added to the dendrimer solution (in citrate-buffered saline (CBS), pH 9.4) and stirred overnight in the dark at room temperature. The solution was then dialyzed against distilled water using a 3.5 kDa dialysis membrane to remove free FITC.

#### FITC-labeled PAMAM-G4-OH dendrimer

Appropriate quantities of the PAMAM-G4-OH dendrimer and FITC were dissolved in anhydrous dimethyl sulfoxide (DMSO). 1-Ethyl-3-(-3-dimethylaminopropyl) carbodiimide (EDC) and 4-dimethylaminopyridine (DMAP) were also dissolved in anhydrous DMSO and added to the PAMAM-G4-OH solution. The reaction mixture stirred for 3 days at room temperature and was then dialyzed against distilled water using a 3.5 kDa dialysis membrane to remove free FITC [2].

#### FITC-labeled PAMAM-G4-COOH dendrimer

To an aqueous solution of dendrimer, an appropriate amount of FITC dissolved in ethanol containing 1 N sodium hydroxide was added, followed by the addition of EDC. The reaction mixture stirred for 72 h, after which the solution was filtered. The solution was then dialyzed against distilled water using 3.5 kDa dialysis membranes to remove free FITC [2].

### The stabilities of the PAMAM-FITC dendrimers at different pH values

The PAMAM-NH_2_-FITC, PAMAM-OH-FITC, and PAMAM-COOH-FITC dendrimers were dialyzed using 3.5 kDa dialysis membranes against pH 6.8 and pH 5.0 PBS for 12 h. Then, the fluorescence intensities of the solutions inside the dialysis membranes were detected at Ex: 495 nm and Em: 519 nm with a fluorescence spectrophotometer (RF-5301(PC)S, Shimadzu, Japan) before and after dialysis.

### Cytotoxicity of the PAMAM dendrimers

The cytotoxicity of the PAMAM dendrimers was detected as previously described [12]. MCF-7/ADR cells were seeded separately in 96-well plates at a density of 6 × 10^3^ cells per well in a humidified atmosphere with 5% CO_2_ at 37 °C and incubated overnight. Then, the medium was replaced with fresh medium containing different concentrations of PAMAM-NH_2_, PAMAM-OH or PAMAM-COOH dendrimers. After incubation for 48 or 72 h, 20 µL of MTT was added, and the cells were incubated for an additional 4 h. The medium was subsequently completely removed, and 150 µL of DMSO was added to dissolve the purple formazan crystals. The absorbance of each well was measured at 490 nm using a microplate reader (Multiskan FC, Thermo Fisher Scientific, USA). Cells without PAMAM dendrimer treatment served as the control group, and the results are expressed as a viability percentage compared to the control cells.

### Time dependence of cellular exocytosis

MCF-7/ADR cells were seeded separately in 6-well culture plates at a density of 1 × 10^6^ cells/well in a humidified atmosphere with 5% CO_2_ for 24 h at 37 °C and then incubated with PAMAM-NH_2_-FITC, PAMAM-OH-FITC, or PAMAM-COOH-FITC dendrimers (20 µg/mL) for 3 h. The cells were rinsed with PBS and immediately incubated with fresh dendrimer-free medium for different lengths of time. Finally, the cells were washed with PBS, resuspended in PBS, and analyzed with a BD FACSCalibur flow cytometer (FACSVerse, BD Biosciences, USA).

### Influence of intracellular transport inhibitors on PAMAM dendrimer exocytosis

MCF-7/ADR cells were seeded separately in 6-well culture plates at a density of 1 × 10^6^ cells/well in a humidified atmosphere with 5% CO_2_ at 37 °C for 24 h. The cells were then incubated with PAMAM-NH_2_-FITC, PAMAM-OH-FITC, or PAMAM-COOH-FITC dendrimers (20 µg/mL) in fresh serum-free medium for 3 h, rinsed three times with cold PBS, and incubated in fresh medium containing various inhibitors (the intracellular transport inhibitors listed in Table 3) for another 3 h. Finally, the cells were washed with PBS, resuspended in PBS, and analyzed with a BD FACSCalibur flow cytometer.

**Table 3:** Inhibitors used in this study, their functions and concentrations

Inhibitor	Function	Concentration
Bafilomycin A1	Inhibitor of endosomal acidification	100 nmol/L
Brefeldin A	Blocks transport from the endoplasmic reticulum to Golgi complex	25 µg/mL
Monensin	Blocks transport from the Golgi complex to plasma membrane	32.5 µg/mL
Verapamil	P-glycoprotein inhibitor	10 µmol/L
Fumitremorgin C	MDR-associated protein inhibitor	5 µmol/L
MK571	Breast cancer resistance protein inhibitor	50 µmol/L

### Influence of ABC protein inhibitors on PAMAM dendrimer exocytosis

MCF-7/ADR cells were seeded separately in 6-well culture plates at a density of 1 × 10^6^ cells/well in a humidified atmosphere with 5% CO_2_ at 37 °C for 24 h. The cells were then incubated with PAMAM-NH_2_-FITC, PAMAM-OH-FITC, or PAMAM-COOH-FITC dendrimers (20 µg/mL) in fresh serum-free medium for 3 h, rinsed three times with cold PBS, and incubated in fresh medium containing ABC protein inhibitors (the ABC protein inhibitors listed in Table 3) for another 3 h. Finally, the cells were washed with PBS, resuspended in PBS, and analyzed with a BD FACSCalibur flow cytometer.

### LDH assay

MCF-7/ADR cells were seeded separately in 96-well plates at a density of 6 × 10^3^ cells per well in a humidified atmosphere with 5% CO_2_ at 37 °C and incubated overnight. Then, the medium was replaced with fresh medium containing different concentrations of PAMAM-NH_2_, PAMAM-OH or PAMAM-COOH dendrimers. After incubation for 1, 3 or 6 h, the culture supernatant was collected and centrifuged. The supernatants were processed according to the LDH analysis kit. The absorbance was measured at 490 nm using a microplate reader.

### PI assay

MCF-7/ADR cells were seeded separately in 6-well culture plates at a density of 1 × 10^6^ cells/well in a humidified atmosphere with 5% CO_2_ for 24 h at 37 °C and then incubated with PAMAM-NH_2_, PAMAM-OH or PAMAM-COOH dendrimers (50 µg/mL) for 1, 3 or 6 h. Next, 20 µL of PI solution (1 mmol/L) was added to 2 mL of each supernatant and incubated for 15 min. Finally, the cells were washed with PBS, resuspended in PBS, and analyzed with a BD FACSCalibur flow cytometer.

### FDA assay

MCF-7/ADR cells were seeded separately in 6-well culture plates at a density of 1 × 10^6^ cells/well in a humidified atmosphere with 5% CO_2_ for 24 h at 37 °C and then incubated with PAMAM-NH_2_, PAMAM-OH or PAMAM-COOH dendrimers (50 µg/mL) for 1, 3 or 6 h. Next, the cells were washed with PBS and suspended in 500 µL of FDA dye working solution (0.5 µmol/L). The cells with dye solution were incubated at 37 °C for 15 min protected from light. Finally, the dye working solution was removed from the cells, and the cells were washed with PBS and resuspended in PBS. Finally, the samples were analyzed with a BD FACSCalibur flow cytometer.

### Organelle colocalization in MCF-7/ADR cells

MCF-7/ADR cells were seeded separately on cover slips in 6-well culture plates (1 × 10^6^ cells/well) in a humidified atmosphere with 5% CO_2_ at 37 °C. Once the cells reached 60–70% confluence, fresh serum-free medium containing PAMAM-NH_2_-FITC, PAMAM-OH-FITC, or PAMAM-COOH-FITC dendrimers (20 µg/mL) was added, followed by incubation for 3 h. After that, the cells were stained with MitoTracker Red (100 nM) for 45 min, washed three times with cold PBS, fixed with 4% formaldehyde, and mounted with Hoechst 33258 (10 µM) at room temperature. Images of the samples were captured by LSCM (FV3000, Olympus, Japan).

### Relative amounts of the PAMAM dendrimers in the mitochondria and cell nuclei

MCF-7/ADR cells were seeded on culture dishes 100 mm in diameter in a humidified atmosphere with 5% CO_2_ at 37 °C. Once the cells reached 90% confluence, fresh serum-free medium containing PAMAM-NH_2_-FITC, PAMAM-OH-FITC, or PAMAM-COOH-FITC dendrimers (20 µg/mL) was added, followed by incubation for 3 h. Then, the cells were washed three times with PBS to remove the PAMAM dendrimers from the cell surface, harvested, centrifuged at 1000 rpm for 5 min and resuspended in PBS. The cell suspension was divided into two parts. The cells in one portion were lysed with lysis buffer containing 0.05% Triton X-100. The other portion of the cell suspension was treated according to the instructions of the cell mitochondria isolation kit or the cell nucleus isolation kit to obtain mitochondria or cell nuclei. Then, the mitochondria or the cell nuclei were lysed by lysis buffer containing 0.05% Triton X-100. Finally, the fluorescence intensities in the cells, mitochondria and cell nuclei were detected at Ex: 495 nm and Em: 519 nm by fluorescence spectrophotometry.

### Influence of MVP on PAMAM dendrimer exocytosis

#### LSCM

MCF-7/ADR cells were seeded separately on cover slips in 6-well culture plates (1 × 10^6^ cells/well) in a humidified atmosphere with 5% CO_2_ at 37 °C for 24 h. Then, the medium was replaced with fresh serum-free Opti-MEM medium. After incubation for 30 min, medium containing siRNA-MVP was added, followed by incubation for 48 h. Then, the cells were washed with PBS and added to fresh serum-free medium containing PAMAM-NH_2_-FITC, PAMAM-OH-FITC, or PAMAM-COOH-FITC dendrimers (20 µg/mL) followed by incubation for 3 h. The cells were rinsed with PBS and immediately incubated with fresh PAMAM dendrimer-free medium for another 3 h. After that, the cells were washed three times with cold PBS, fixed with 4% formaldehyde, and mounted with DAPI (0.1 µg/mL) at room temperature. Images of the samples were captured by LSCM.

#### Quantitative determination

MCF-7/ADR cells were seeded on culture dishes 100 mm in diameter in a humidified atmosphere with 5% CO_2_ at 37 °C for 24 h. Then, the medium was replaced with fresh serum-free Opti-MEM medium. After incubation for 30 min, medium containing siRNA-MVP was added, followed by incubation for 48 h. Then, the cells were washed with PBS and added to fresh serum-free medium containing PAMAM-NH_2_-FITC, PAMAM-OH-FITC, or PAMAM-COOH-FITC dendrimers (20 µg/mL) followed by incubation for 3 h. The cells were rinsed with PBS and immediately incubated with fresh PAMAM dendrimer-free medium for another 3 h. After that, the cells were washed three times with PBS to remove the PAMAM dendrimers from the cell surface, harvested, centrifuged at 1000 rpm for 5 min and resuspended in PBS. The cell suspension was divided into two parts. The cells in one portion were lysed with lysis buffer containing 0.05% Triton X-100. The other portion of the cell suspension was treated according to the instructions of the cell nucleus extraction kit to obtain the cell nucleus, and then the nuclei were lysed with lysis buffer containing 0.05% Triton X-100. Finally, the fluorescence intensities in the cells and cell nuclei were detected at Ex: 495 nm and Em: 519 nm by fluorescence spectrophotometry.

### Statistical analysis

All values are reported as the mean ± SD. The data were analyzed statistically by one-way analysis of variance and Student’s t-test using SPSS 13.0 software. A *P* value < 0.05 and a *P* value < 0.01 indicate statistically significant differences.

## Supplementary Information

**Additional file 1.** Additional figures and tables.

## Data Availability

The datasets used and/or analyzed during the current study are available from the corresponding author upon reasonable request.

## References

[1] Palmerston Mendes L, Pan J, Torchilin VP (2017). Dendrimers as nanocarriers for nucleic acid and drug delivery in cancer therapy. Molecules.

[2] Perumal OP, Inapagolla R, Kannan S, Kannan RM (2008). The effect of surface functionality on cellular trafficking of dendrimers. Biomaterials.

[3] Li J, Liang H, Liu J, Wang Z (2018). Poly (amidoamine) (PAMAM) dendrimer mediated delivery of drug and pDNA/siRNA for cancer therapy. Int J Pharm.

[4] Tai Z, Ma J, Ding J, Pan H, Chai R, Zhu C (2020). Aptamer-functionalized dendrimer delivery of plasmid-encoding lncRNA MEG3 enhances gene therapy in castration-resistant prostate cancer. Int J Nanomed.

[5] Patra JK, Das G, Fraceto LF, Campos EVR, Rodriguez-Torres MDP, Acosta-Torres LS (2018). Nano based drug delivery systems: recent developments and future prospects. J Nanobiotechnol.

[6] Tariq I, Ali MY, Janga H, Ali S, Amin MU, Ambreen G (2020). Downregulation of MDR 1 gene contributes to tyrosine kinase inhibitor induce apoptosis and reduction in tumor metastasis: A gravity to space investigation. Int J Pharm.

[7] Han M, Lv Q, Tang XJ, Hu YL, Xu DH, Li FZ (2012). Overcoming drug resistance of MCF-7/ADR cells by altering intracellular distribution of doxorubicin via MVP knockdown with a novel siRNA polyamidoamine-hyaluronic acid complex. J Control Release.

[8] Kesharwani P, Jain K, Jain NK (2014). Dendrimer as nanocarrier for drug delivery. Prog Polym Sci.

[9] Sadekar S, Ghandehari H (2012). Transepithelial transport and toxicity of PAMAM dendrimers: implications for oral drug delivery. Adv Drug Deliv Rev.

[10] Leroueil PR, Hong S, Mecke A, Orr JRB, Holl BG (2007). Nanoparticle interaction with biological membranes: does nanotechnology present a Janus face? Acc. Chem Res.

[11] Leroueil PR, Berry SA, Duthie K, Han G, Rotello VM, McNerny DQ (2008). Wide varieties of cationic nanoparticles induce defects in supported lipid bilayers. Nano Lett.

[12] Zhang J, Liu D, Zhang M, Sun Y, Zhang X, Guan G (2016). The cellular uptake mechanism, intracellular transportation, and exocytosis of polyamidoamine dendrimers in multidrug-resistant breast cancer cells. Int J Nanomedicine.

[13] Zeng Y, Kurokawa Y, Win-Shwe T-T, Zeng Q, Hirano S, Zhang Z (2016). Effects of PAMAM dendrimers with various surface functional groups and multiple generations on cytotoxicity and neuronal differentiation using human neural progenitor cells. J Toxicol Sci.

[14] Saraswathy M, Gong S (2013). Different strategies to overcome multidrug resistance in cancer. Biotechnol Adv.

[15] Patel NR, Pattni BS, Abouzeid AH, Torchilin VP (2013). Nanopreparations to overcome multidrug resistance in cancer. Adv Drug Deliv Rev.

[16] Gao Z, Zhang L, Sun Y (2012). Nanotechnology applied to overcome tumor drug resistance. J Control Release.

[17] Park H, Saravanakumar G, Kim J, Lim J, Kim WJ (2020). Tumor microenvironment sensitive nanocarriers for bioimaging and therapeutics. Adv Healthc Mater.

[18] Wang Y, Zhou K, Huang G, Hensley C, Huang X, Ma X (2014). A nanoparticle-based strategy for the imaging of a broad range of tumours by nonlinear amplification of microenvironment signals. Nat Mater.

[19] Oddone N, Lecot N, Fernandez M, Rodriguez-Haralambides A, Cabral P, Cerecetto H (2016). In vitro and in vivo uptake studies of PAMAM G4.5 dendrimers in breast cancer. J Nanobiotechnol.

[20] Seib FP, Jones AT, Duncan R (2007). Comparison of the endocytic properties of linear and branched PEIs, and cationic PAMAM dendrimers in B16f10 melanoma cells. J Control Release.

[21] Desoize B, Jardillier J (2000). Multicellular resistance: a paradigm for clinical resistance?. Crit Rev Oncol Hematol.

[22] Chai GH, Hu FQ, Sun J, Du YZ, You J, Yuan H (2014). Transport pathways of solid lipid nanoparticles across Madin-Darby canine kidney epithelial cell monolayer. Mol Pharm.

[23] SG M, H-PH LC (1992). Post-Golgi membrane traffic: brefeldin A inhibits export from distal Golgi compartments to the cell surface but not recycling. J Cell Biol.

[24] Kuismanen E, Saraste J, Pettersson RF (1985). Effect of monensin on the assembly of Uukuniemi virus in the Golgi complex. J Virol.

[25] Kathawala RJ, Gupta P, Ashby CR, Chen ZS (2015). The modulation of ABC transporter-mediated multidrug resistance in cancer: a review of the past decade. Drug Resist Updat.

[26] W VG, W-R U IKHS (1995). The leukotriene LTC4 receptor antagonist MK571 specifically modulates MRP associated multidrug resistance. Biochem Biophys Res Commun.

[27] Av L, JD A, K G-J. AHS (2001). Inhibition of BCRP-Mediated drug efflux by fumitremorgin-type indolyl diketopiperazines. Bioorg Med Chem Lett.

[28] Hong S, Bielinska AU, Mecke A, Keszler B, Beals JL, Shi X (2004). Interaction of poly(amidoamine) dendrimers with supported lipid bilayers and cells: hole formation and the relation to transport. Bioconjug Chem.

[29] Umebayashi Y, Miyamoto Y, Wakita M, Kobayashi A, Nishisaka T (2003). Elevation of plasma membrane permeability on laser irradiation of extracellular latex particles. J Biochem.

[30] Mao H, Tao T, Song D, Liu M, Wang X, Liu X (2016). Zedoarondiol inhibits platelet-derived growth factor-induced vascular smooth muscle cells proliferation via regulating AMP-activated protein kinase signaling pathway. Cell Physiol Biochem.

[31] Gang X, Qian W, Zhang T, Yang X, Xia Q, Cheng D (2017). Aurora B kinase is required for cell cycle progression in silkworm. Gene.

[32] Mi XG, Song ZB, Sun LG, Bao YL, Yu CL, Wu Y (2016). Cardamonin inhibited cell viability and tumorigenesis partially through blockade of testes-specific protease 50-mediated nuclear factor-kappaB signaling pathway activation. Int J Biochem Cell Biol.

[33] Sakhtianchi R, Minchin RF, Lee KB, Alkilany AM, Serpooshan V, Mahmoudi M (2013). Exocytosis of nanoparticles from cells: role in cellular retention and toxicity. Adv Colloid Interface Sci.

[34] Torchilin VP (2006). Recent approaches to intracellular delivery of drugs and DNA and organelle targeting. Annu Rev Biomed Eng.

[35] Chen LB (1988). Mitochondrial membrane potential in living cells. Annu Rev Cell Biol.

[36] Brunner S, Sauer T, Carotta S, Cotten M, Saltik M, Wagner E (2000). Cell cycle dependence of gene transfer by lipoplex, polyplex and recombinant adenovirus. Gene Ther.

[37] van Zon A, Mossink MH, Houtsmuller AB, Schoester M, Scheffer GL, Scheper RJ (2006). Vault mobility depends in part on microtubules and vaults can be recruited to the nuclear envelope. Exp Cell Res.

[38] Kitchens KM, Foraker AB, Kolhatkar RB, Swaan PW, Ghandehari H (2007). Endocytosis and interaction of poly (amidoamine) dendrimers with Caco-2 cells. Pharm Res.

