# Supplementary material for: Effects of the surface charge of polyamidoamine dendrimers on cellular exocytosis and the exocytosis mechanism in multidrug-resistant breast cancer cells
Source: J Nanobiotechnology. 2021 May 12;19:135. doi: 10.1186/s12951-021-00881-w (PMC8114490; doi:10.1186/s12951-021-00881-w)
Supplement: Supplementary file 1 — Additional file 1. Additional figures and tables. [file 12951_2021_881_MOESM1_ESM.doc]

# Additional file

**Extracellular binding of PAMAM dendrimers**

We detected the cellular uptake rates of PAMAM-NH_2_, PAMAM-OH and PAMAM-COOH dendrimers in MCF-7/ADR cells at 4 °C (Fig. S1). Fig. S1 showed that the uptake rates of PAMAM-NH_2_, PAMAM-OH and PAMAM-COOH dendrimers were always below 5%, revealing that the extracellular binding of PAMAM dendrimers at the MCF-7/ADR cell surface could be removed through the cell sample treatment method in this study, and the intracellular fluorescence intensity tested represents the intracellular content of PAMAM dendrimers.


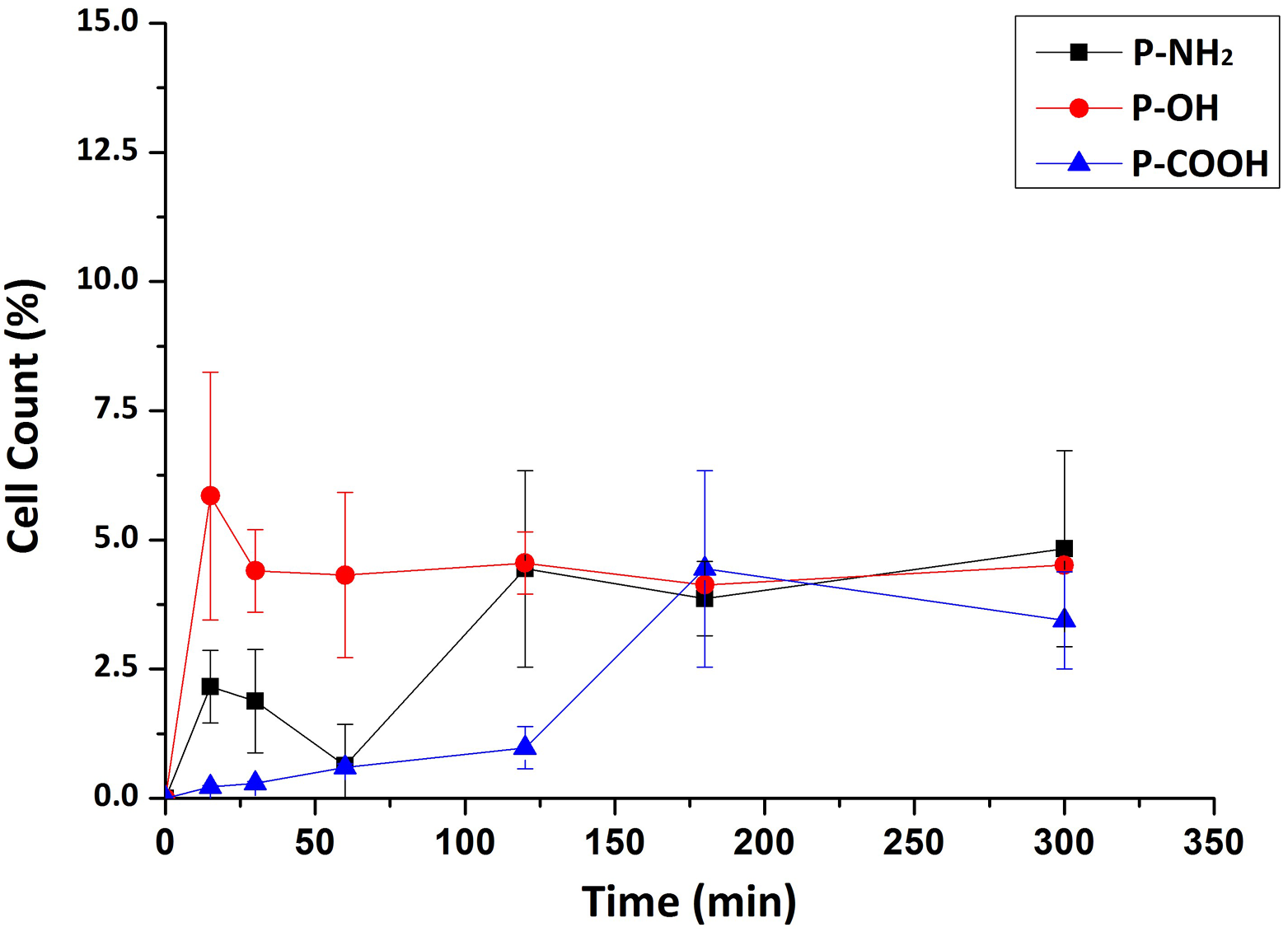


**Fig. S1** The amount of PAMAM dendrimers binding at the MCF-7/ADR cells surface (mean ± SD, n=3).

**RI assay**

The cells viabilities at different concentrations of doxorubicin (DOX) were used to investigate the cytotoxicty in vitro. As shown in Fig. S2, the viabilities of MCF-7/ADR cells were higher than that of MCF-7 cells at 48h, which showed that DOX exhibited greater cytotoxicity in MCF-7 cells than that in MCF-7/ADR cells. RI represented the multidrug resistance of tumor cells. RI = IC50(MCF-7/ADR）/ IC50(MCF-7). As shown in Table S1, the IC50 of DOX against the MCF-7/ADR cells was higher than that against the MCF-7 cells, and the RI was 51.75, which suggested that the MCF-7/ADR cells were resistant to DOX.

**
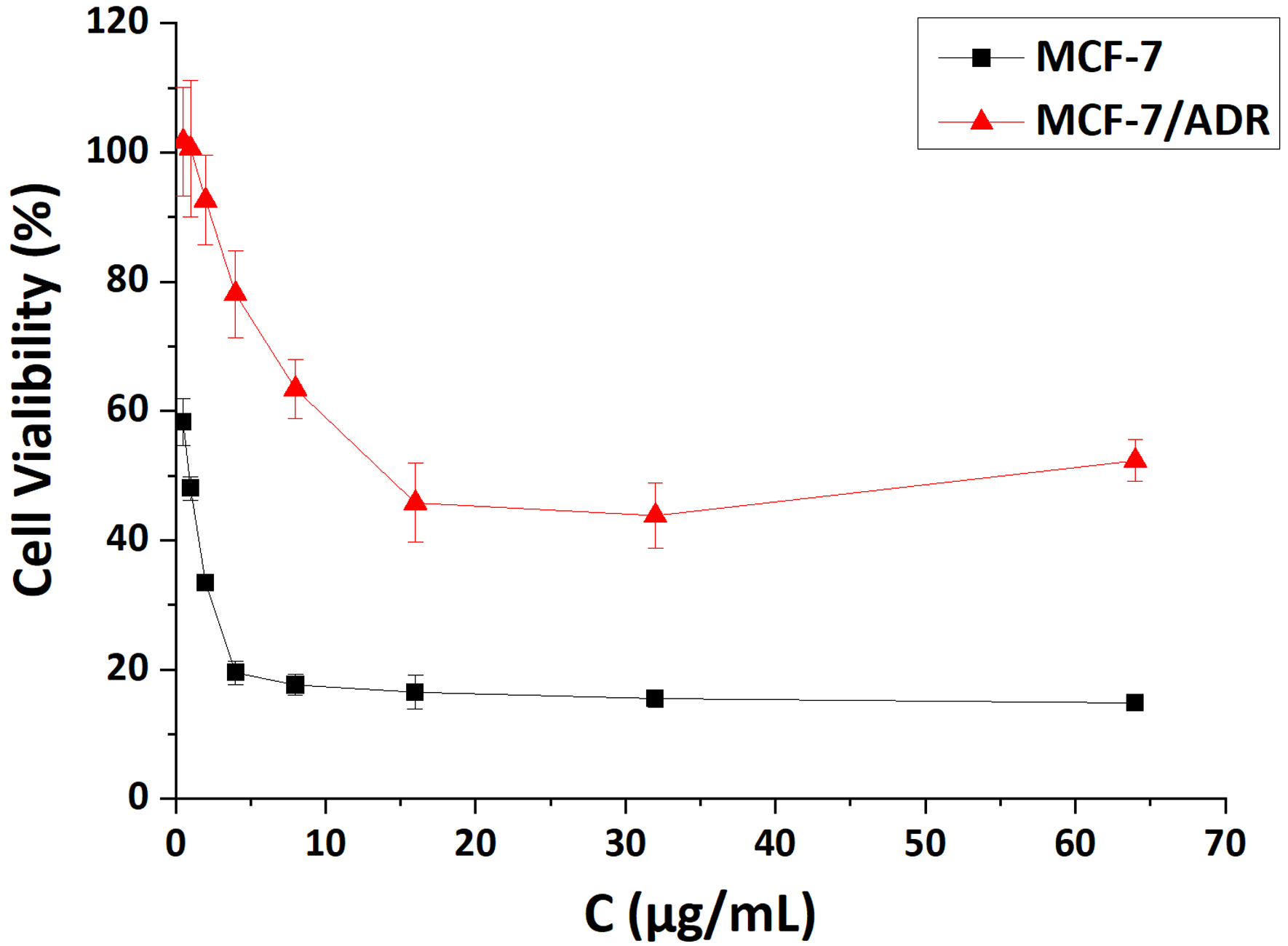
**

**Fig. S2** In vitro cytotoxicity of DOX at different concentrations against MCF-7 and MCF-7/ADR cells for 48 h (mean ± SD, n = 6).

**Table S1 IC50 value (μg/mL) and RI of DOX in cells**

|  | IC50 | | Resistance Index  (RI) |
| --- | --- | --- | --- |
|  | MCF-7 | MCF-7/ADR |
| DOX | 0.567 | 29.34 | 51.75 |
